# Natural History of DNA-Dependent DNA Polymerases: Multiple Pathways to the Origins of DNA

**DOI:** 10.3390/v15030749

**Published:** 2023-03-14

**Authors:** Sávio Torres de Farias, Ariadne Nobrega Marinho Furtado, Ariosvaldo Pereira dos Santos Junior, Marco V. José

**Affiliations:** 1 Departamento de Biologia Molecular, Universidade Federal da Paraíba, João Pessoa 58051-900, Brazil; 2 Network of Researchers on the Chemical Evolution of Life (NoRCEL), Leeds LS7 3RB, UK; 3 Theoretical Biology Group, Instituto de Investigaciones Biomédicas, Universidad Nacional Autónoma de México, Ciudad de México C.P. 04510, Mexico

**Keywords:** DNA origin, viruses’ evolution, cell evolution

## Abstract

One of the major evolutionary transitions that led to DNA replacing RNA as the primary informational molecule in biological systems is still the subject of an intense debate in the scientific community. DNA polymerases are currently split into various families. Families A, B, and C are the most significant. In bacteria and some types of viruses, enzymes from families A and C predominate, whereas family B enzymes are more common in Archaea, Eukarya, and some types of viruses. A phylogenetic analysis of these three families of DNA polymerase was carried out. We assumed that reverse transcriptase was the ancestor of DNA polymerases. Our findings suggest that families A and C emerged and organized themselves when the earliest bacterial lineages had diverged, and that these earliest lineages had RNA genomes that were in transition—that is, the information was temporally stored in DNA molecules that were continuously being produced by reverse transcription. The origin of DNA and the apparatus for its replication in the mitochondrial ancestors may have occurred independently of DNA and the replication machinery of other bacterial lineages, according to these two alternate modes of genetic material replication. The family C enzymes emerged in a particular bacterial lineage before being passed to viral lineages, which must have functioned by disseminating this machinery to the other lineages of bacteria. Bacterial DNA viruses must have evolved at least twice independently, in addition to the requirement that DNA have arisen twice in bacterial lineages. We offer two possible scenarios based on what we know about bacterial DNA polymerases. One hypothesis contends that family A was initially produced and spread to the other lineages through viral lineages before being supplanted by the emergence of family C and acquisition at that position of the principal replicative polymerase. The evidence points to the independence of these events and suggests that the viral lineage’s acquisition of cellular replicative machinery was crucial for the establishment of a DNA genome in the other bacterial lineages, since these viral lineages may have served as a conduit for the machinery’s delivery to other bacterial lineages that diverged with the RNA genome. Our data suggest that family B initially established itself in viral lineages and was transferred to ancestral Archaea lineages before the group diversified; thus, the DNA genome must have emerged first in this cellular lineage. Our data point to multiple evolutionary steps in the origins of DNA polymerase, having started off at least twice in the bacterial lineage and once in the archaeal lineage. Given that viral lineages are implicated in a significant portion of the distribution of DNA replication equipment in both bacterial (families A and C) and Archaeal lineages (family A), our data point to a complex scenario.

## 1. Introduction

Several transitions that occurred throughout the origin and maturation of biological systems were crucial for the development of life as we know it today. Some of the events that enabled life to exist on our planet include the emergence of the first informational molecule, the structuring of the genetic code, the formation of the translation system, and the establishment of basal metabolism. Currently, it is practically a consensus in the scientific community that the first informational molecule to be fixed in biological systems was RNA [1,2,3,4]. Despite being extremely versatile, RNA was replaced during the evolutionary process by DNA in cellular lineages, as well as in several viral groups. There is still a great deal of discussion in the scientific community about the processes that resulted in the transition from RNA to DNA as the primary informational molecule [5,6,7,8,9,10]. Some hypotheses suggest that this transition occurred before the diversification of the basal cellular lineages, Bacteria and Archaea, in a lineage already having cell structure, known as LUCA (last universal common ancestor) [11,12]. According to this hypothesis, DNA appeared only once in the history of living beings and was inherited by cellular lineages from a common ancestor. However, other hypotheses suggest that LUCA still had an RNA genome and that after the diversification of the basal lineages, these independently replaced the RNA molecule for DNA as the main informational molecule [7,8,13]. By this hypothesis, the DNA molecule would have multiple origins in cellular lineages. According to other theories, a cellularized LUCA could not have existed because the basal lineages descended from a group of molecular systems called progenotes, which had RNA as its information storage medium. As a result, the transition from RNA to DNA must have happened independently in bacterial and archaeal cells after their establishment [14,15,16,17,18]. These hypotheses are in line with hypotheses that suggest that the basal cellular lineages had independent origins [18,19]. Among the evidence that supports the idea that the basal cell lines originated independently and with an RNA genome is the fact that the membrane structures of bacteria and archaea are different. There is no shared structure or synthesis pathway [18]. More importantly, the enzymes that act on DNA metabolism do not have homology between these lineages [5,6,7,8]. In addition to this evidence, we also have the fact that thymidylate synthase, the enzyme involved in the synthesis of thymine, has two versions, one in Archaea and the other in Bacteria, thereby reinforcing the idea of an independent origin of DNA in these lineages [8]. Even when significant evidence points to the independent emergence of DNA in basal cellular lineages, some studies support the hypothesis that DNA had already begun to establish itself in viral lineages at the same time as the earliest cellular lineages [8,20]. Thus, some hypotheses suggest that the machinery for processing biological information in the DNA of cells was inherited by the horizontal transfer from these viral lineages [8,20]. According to some authors, the conversion of RNA to DNA in cells took place because of an infection by a retrovirus, which used its replication system to turn the RNA already present in the cells into DNA with the help of a reverse transcriptase. As a result, the DNA polymerases independently established themselves in each of the basal cellular lineages [6,8,10,18]. Some studies indicate that the different DNA polymerases originated from reverse-transcriptase-type enzymes, and that after their origins, they followed independent roads of diversification, which would explain how homology was lost in the different DNA polymerase families [21]. Currently, DNA polymerases are divided into several families. The most important are families A, B, and C. Enzymes from families A and C are predominantly present in bacteria and some groups of viruses; and family B enzymes prevail in Archaea, Eukarya, and some groups of viruses [22]. Despite not showing clear homology in terms of sequence, all polymerases share structural similarities. It is possible to identify at least three subdomains in all of them—the palm subdomain, fingers subdomain, and thumb subdomain. The palm subdomain is where we find the catalytic site; in the fingers subdomain, the interaction between the template and the nucleotides occurs during polymerization; and the thumb subdomain is the most versatile and is involved in the fidelity of the template reading, in addition to in the affinity of the enzyme for the template [23]. In this sense, although we do not observe clear homology, we think that these molecules have deep homology, as they must share the same ancestral molecule, and that during the process of diversification, which occurred by radiation from a reverse-transcriptase-type protein, the homology was lost.

In the present work, we assume that reverse transcriptases are the ancestors of DNA polymerases, and we try to explain how DNA polymerases evolved and became diverse in both viral and basal cellular lineages.

## 2. Materials and Methods

### 2.1. Obtaining the Analyzed Sequences

In the present work, proteins from families A, B, and C of DNA-dependent DNA polymerases were analyzed. Sequences were obtained from GenBank in the Refseq database. For family A DNA-dependent DNA polymerases, 100 sequences were obtained—80 representing Bacteria and chloroplast/mitochondria and 20 representing viruses. For family B of DNA-dependent DNA polymerases, 72 sequences were obtained,—6 from eukaryotes, 20 from archaea, 6 from bacteria, and 20 from viruses. For family C of DNA-dependent DNA polymerases, 61 sequences were obtained—51 from bacteria and 10 from viruses. As an outgroup, 6 RNA-dependent DNA polymerase sequences were used. All used sequences are curated and available in the Supplementary Material.

### 2.2. Alignment and Phylogeny

Alignments were generated using MAFFT version 7 [24] according to default criteria. ML analyses were performed using the software RAxML version 8.2.10 [25], which is available on the CIPRES portal [26] (http://www.phylo.org/). The analysis first involved 100 ML searches, each starting from one randomized stepwise addition parsimony tree (command –f d), under a GTRGAMMA model. All other parameters were estimated by the software. To assess the reliability of the nodes, nonparametric bootstrapping replicates under the same model were computed, allowing the program to halt bootstrapping automatically with the autoMRE bootstrapping criterion. To plot the calculated bootstrap values on the branches, the command –fb was used. The original trees are available in the Supplementary Material.

## 3. Results and Discussion

### On the Origin of DNA-Dependent DNA Polymerase

One of the major evolutionary transitions that led to DNA replacing RNA as the primary informational molecule in biological systems is still the subject of a long debate in the scientific community [5,6,7,8]. According to some evidence, an RNA-dependent RNA polymerase was the first polymerase to appear when RNA was the primary molecule in genomes [27]. In this manner, the transition event of the informational molecule in biological systems was made possible by the diversification of this first polymerase into another group of polymerases, the RNA-dependent DNA polymerase, during the processes that led to the exchange from RNA to DNA [21]. RNA-dependent DNA polymerases work by converting an RNA molecule into double-stranded DNA in three steps. Initially, these enzymes make a copy of DNA from an RNA molecule, then degrade the RNA molecule, and finally, synthesize the complementary strand of DNA, obtaining at the end of the process a double-stranded DNA. The emergence of RNA-dependent DNA polymerase aided in the process of transition from RNA to DNA as the primary informational molecule. However, its activity is restricted, and numerous mutations are inserted in the process, since this enzyme lacks error-correcting mechanisms [28]. As a result, the selective pressure brought on by the switch from RNA to DNA caused the RNA-dependent DNA polymerases to diversify into a class of specialized enzymes which had an error-correcting system that allowed maturation and increased genome size in early cellular lineages—the DNA-dependent DNA polymerases. It is intriguing to observe how the DNA-dependent DNA polymerase and the RNA-dependent DNA polymerases are comparable structurally, although we cannot detect similarity in terms of primary sequence [23]. Another point to keep in mind is that some DNA-dependent DNA polymerases exhibit some reverse transcription activity and that only a few mutations can induce this activity to emerge in DNA-dependent DNA polymerases [29,30,31]. These facts, when taken together, may suggest that these two groups of proteins have a common ancestor. The lack of detectable homology between the various families, however, raises the possibility that the events that led to the maturation of these lineages may have occurred independently. 

In this sense, by examining the pattern of diversification to DNA-dependent DNA polymerases, we may infer the routes followed by cellular lineages during the process of transition from RNA to DNA as the major informational molecule.

## 4. Bacterial DNA-Dependent DNA Polymerase

### 4.1. Family A

**Figure 1:**
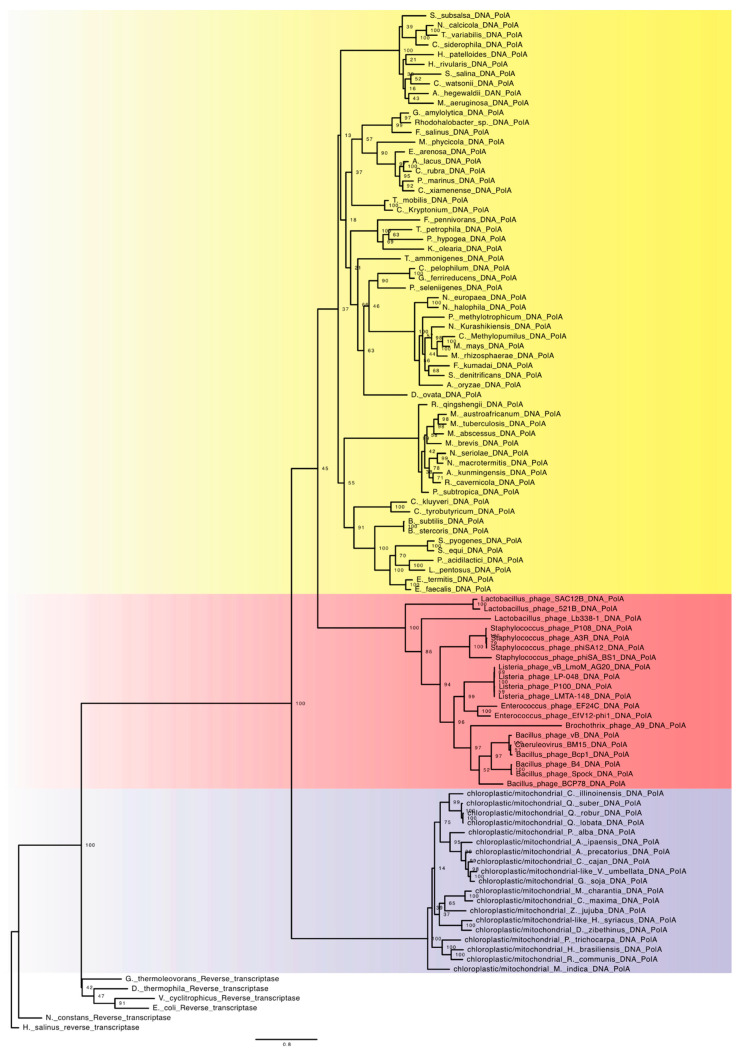
ML tree of family A DNA-dependent DNA polymerases. In blue, mitochondria/chloroplasts. In red, viruses, and in yellow, bacteria.

The family A DNA-dependent DNA polymerases are widely distributed in the Bacteria domain, and in mitochondria and chloroplasts. Like many polymerases, it has a general structure organized like a right hand, with palm, finger, and thumb structural domains [23]. In bacteria, family A is involved in some repair processes, and in the removal and maturation of Okazaki fragments. In mitochondria and chloroplasts, this family is involved in genome replication [22,32]. In our analysis, sequences of family A proteins from different groups of bacteria were included, along with polymerases from viruses, mitochondria, and chloroplasts having RNA-dependent DNA polymerases as their root. The results can be seen in Figure 1. It is interesting that the first group which diversified within family A were the mitochondria/chloroplast polymerases, so we observed an older branch that precedes the branch of bacteria, formed by viral lineages. The results suggest that the family A emerged and structured itself when the first lineages of bacteria had differentiated, suggesting that these initial lineages had an RNA genome or a genome in transition, where the information would have been stored temporally in the DNA molecule that was constantly formed by reverse transcription. 

The emergence of family A DNA polymerases in mitochondria/chloroplast ancestors allowed the maturation and fixation of DNA as an informational molecule in this lineage. It should be noted that when we talk about the ancestors of mitochondria and chloroplasts, we are not referring directly to the more recent group that gave rise to these organelles, but to the ancestral lineage of bacteria that gave rise to the group that would later establish endosymbiosis. During this fixation process or after the maturation of DNA to being the main informational molecule in the ancestors of mitochondria, this machinery must have been transferred to viral lineages, which must have allowed the maturation of the first lineages of bacterial viruses with DNA as the informational molecule, and subsequently, the family A proteins spread to the rest of the groups of bacteria which still had RNA genomes.. In this context, it is worth mentioning that although the scientific community is widely aware that mitochondria and chloroplasts originated from bacterial lineages, when we observe the DNA replication pattern of bacteria and mitochondria/chloroplasts, we can identify significant differences. Although both systems need a primer for the polymerase to start its activity, in mitochondria and chloroplasts, the two DNA strands are replicated independently. Replication is initiated in the heavy strand in a single direction, and after reaching the origin of replication of the light strand, this strand replicates in the opposite direction [33]. In bacteria, the two strands of DNA replicate bidirectionally. Both strands of DNA are replicated at the same time. The replication of the two DNA strands in a bidirectional way requires the formation of multiple Okazaki fragments on the discontinuous strand, which must be matured at the end of the process, whereas in the replication of the mitochondrial genome, these structures do not form: replication is started by just one primer on each of the strands. These two alternative forms of replication of genetic material may indicate that in the ancestors of mitochondria, the origins of DNA and the replication machinery of this genetic material occurred independently of those of DNA and the replication machinery of other bacterial lineages. This notion is reinforced by the presence of another family of polymerases involved in genome replication in these bacterial lineages.

### 4.2. Family C

**Figure 2:**
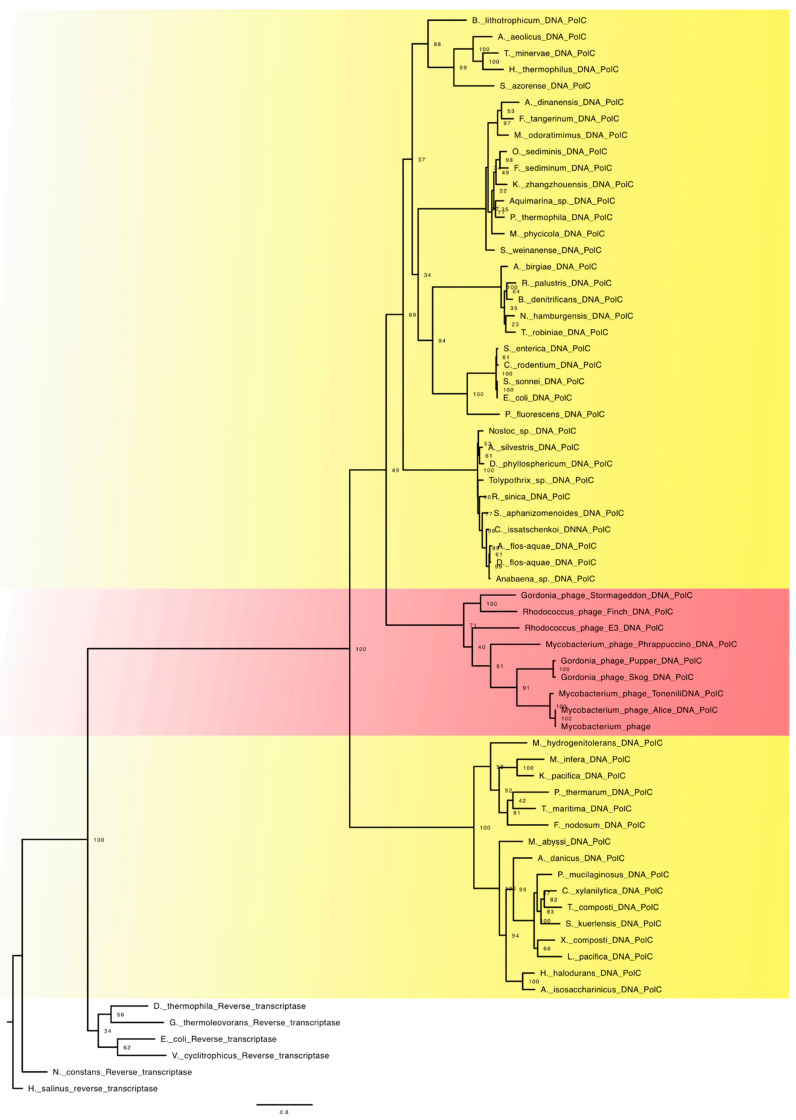
ML tree of family C DNA-dependent DNA polymerases. In red are the viruses, and in yellow are the bacteria.

Family C of DNA-dependent DNA polymerases is widely distributed in Bacteria and some viral families. In these groups, this family of polymerases is responsible for genome replication in both continuous and discontinuous strands. As mentioned above, the bacterial genome replication model—bidirectional and replication of both strands at the same time—generates, on the discontinuous strand, a series of RNA and DNA heteroduplex regions, known as Okazaki fragments [34]. At the conclusion of the procedure, these heteroduplex sections must be repaired by deleting the RNA sequences and filling these regions with DNA [35]. Figure 2 shows the phylogeny of family C DNA polymerases. It is intriguing that the trend of diversification follows a similar path to that seen in Family A. The extremophile bacteria and bacteria from Firmicutes phylum were the first group in this scenario to diversify, then viral lineages, and lastly the other bacterial lineages. When we say that the pattern is comparable, we mean that similarly to the family A enzymes, the family C enzymes emerged in a particular bacterial lineage before being passed to viral lineages, which must have functioned by disseminating this machinery to the other lineages of bacteria.

This resemblance in patterns of diversification supports the hypothesis that the first bacterial lineages were generated with an RNA or transitional genome, as previously noted, and that DNA was merely a transient stage that was continually being recreated through reverse transcription [4,5,6,7,8,18,19]. The idea that the transition from RNA to DNA as the primary informational molecule occurred at least twice independently in the bacterial lineage is supported by the absence of family C in mitochondrial lineages, the differentiated pattern of genetic material replication, and the appearance of family C DNA polymerases in a particular group of bacteria. 

We can offer two possible scenarios based on what we know about bacterial DNA polymerases. The first hypothesis contends that family A was initially produced and spread to the other lineages through viral lineages before being supplanted by the emergence of family C and the acquisition at that position of the principal replicative polymerase.

In this scenario, due to the low progressivity of family A DNA polymerases, the acquisition of the family C polymerases allowed greater efficiency in genome replication, along with an increase in its size. Family A was co-opted for a secondary function involved in the resolution of the Okazaki fragments. In the second scenario, family C DNA polymerases were acquired first and spread via viral lineages to other bacterial lineages. In this scenario, in these initial lineages, Okazaki fragments could generate RNA and DNA hybrid points that could not be resolved by the replicative enzyme, and a reverse transcriptase could be used to mature these regions. Due to the family A polymerases’ ability to resolve these heteroduplex regions, the secondary acquisition of these enzymes allowed for the improvement of the replication system. It is worth mentioning that in family A DNA polymerases, residual activity of reverse transcription has already been described, which suggests that an ancestral vestige is maintained in this family, which must have enabled this family to replace a function performed by reverse transcriptase [29,31]. The elimination of reverse transcriptase in bacterial systems during the replication process may have occurred to avoid competition between this group of polymerases and the DNA polymerases that emerged in the lineages that underwent the transition from RNA to DNA in the genome. Note that not only must DNA have arisen twice in bacterial lineages, but bacterial DNA viruses must also have emerged at least twice independently.

## 5. Archaeal and Eukaryotic DNA-Dependent DNA Polymerase

### Family B

**Figure 3:**
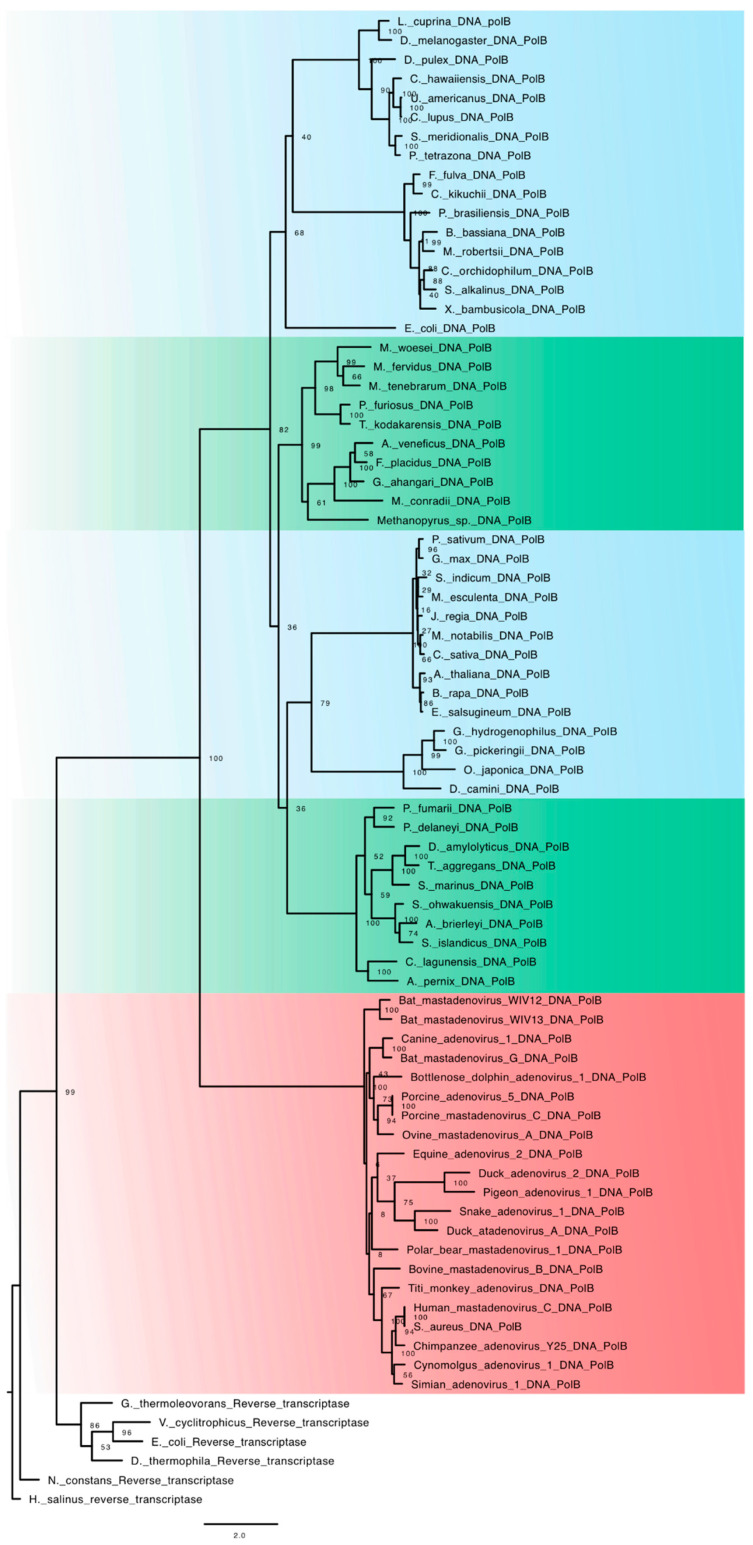
ML tree of family B DNA-dependent DNA polymerases. In red, viruses. In green, archaea, and in blue, eukaryotes.

Family B DNA-dependent DNA polymerases are widely distributed among organisms of the Archaea and Eukarya lineages, and in eukaryotes, they are further diversified into several groups [36]. The DNA polymerases of this family are involved in several processes. In the process of replicating the genetic material, they act both in the replication of the genome and in the maturation of the Okazaki fragments, showing different functional versatility of the bacterial polymerases, since in this lineage, there was a specialization of the polymerases C and A for replication and maturation of Okazaki fragments, respectively. Some studies have shown that with a few mutation points, specimens of this family acquired a reverse transcriptase function, which may indicate an evolutionary reversion process, since it is suggested that all families of DNA-dependent DNA polymerases may have had a reverse transcriptase as an ancestral molecule [30]. In our analysis, sequences from the main groups of Archaea and Eukarya, and viral lineages, were used. The result can be seen in Figure 3. This pattern of diversification differs from the patterns shown in families A and C. The DNA polymerases found in viruses are the first lineage in family B to diversify, followed by the diversity in cellular lineages. 

This unique pattern of diversification shows that the emergence of DNA as an informational molecule in Archaea and Eukarya had a separate evolutionary trajectory from the evolutionary history of the maturation of the DNA in bacterial lineages. In this sense, we conceive a scenario in which the Archaeal lineage was initially established with an RNA genome, and that, prior to the diversification of the various groups, the conversion of this RNA genome to DNA took place via the acquisition of viral machinery for DNA replication. In this context, Archaeal viruses’ lineages acquired their DNA genome before cellular lineages. It should be noted that, in this scenario, DNA as an informational molecule must have emerged before this same process occurred in bacterial lineages. Thus, not only did DNA originate independently in basal cellular lineages, but it also occurred at distinct temporal stages.

## 6. Viral DNA-Dependent DNA Polymerase

Many hypotheses have been proposed about the origin and diversification of viral lineages. Models for their emergence assume an origin from three scenarios: (i) virus first, (ii) cell reduction, and (iii) escape [20,37,38,39,40]. Although these models suggest that these hypotheses are exclusive, upon a deeper analysis, they show themselves as alternative but compatible models, in that the assumption of a model for a certain group of viruses does not invalidate the possibility of other models being applied to other viral groups. Our data do not allow us to make inferences about the origin of viruses, but from them, we can delineate routes for the emergence of groups of viruses that had DNA as an informational molecule, both from viruses to bacteria lineages and from viruses to the Archaea and Eukarya lineages. From the results obtained, we can identify at least three routes for the emergence of viruses with DNA genomes. For viruses of bacterial lineages, the data suggest that at least two routes were followed in this process of transition and maturation of the viral DNA genome. The first one suggested is the acquisition of the replicative material from the ancestral lineage of mitochondria by the capturing of a family A DNA polymerase, and the second one is the capturing of the family C DNA polymerases. The data suggest that these events were independent and that the acquisition of cellular replicative machinery by the viral lineage was important for the consolidation of a DNA genome in the other bacterial lineages, since these viral lineages may have acted as a delivery system for this machinery to other bacterial lineages that diversified with RNA genomes. In this context, since viral lineages form a separate clade, it is more parsimonious to think that these lineages emerged contemporaneously with the emergence of bacterial lineages and not through an escape process because if it were through an escape process, we should be able to identify viral lineages spread into bacterial clades following a pattern of diversification similar to that of the lineages from which they originated. These data indicate that viruses and bacteria have established a co-evolutionary process since their origins, with an intense exchange of genetic material which enabled important transitions for both groups. 

On the other hand, when analyzing the Archaeal viral lineages, we observed a slightly different pattern, since the data suggest that these lineages acquired their DNA genome before the emergence of DNA in cellular lineages. In this context, viral lineages transferred their replicative machinery to basal Archaeal lineages before the initial diversification of this group. In this way, this transfer process at a very primitive stage for Archaeal lineages may have provided the maturation of DNA as an informational molecule before this process appeared in bacterial lineages. Altogether, our data suggest a complex scenario for the emergence of DNA in different lineages of organisms, with this process following at least three independent routes.

## 7. Last Considerations

### Proposal of a Scenario for the Emergence of the DNA Genome in Cellular Lineages

The origin of DNA as an informational molecule in cellular lineages represents a huge evolutionary novelty, since it enabled greater stability in the storage of biological information, and it allowed an increase in the size of the genome of organisms. In the present work, the data suggest multiple origins for this molecule, having originated at least twice in the bacterial lineage and once in the Archaeal lineage. These data suggest a complex scenario, as it is suggested that viral lineages played an important role in the dissemination of DNA replication machinery in both bacterial and archaeal lineages. From the data presented, we can infer a scenario for the emergence of DNA as the main informational molecule in cellular lineages, as well as in viral lineages. Before suggesting a scenario for the origin of a DNA genome in basal cellular lineages, as well as for some viral lineages, we must delve into the scenario prior to this transitional event. In this context, we can think of three scenarios before the diversification of basal lineages: (i) the last common universal ancestor was a cellular organism with a DNA genome; (ii) the last common universal ancestor was cellularized and had an RNA genome, and (iii) the last common universal ancestor was not cellularized, thereby being a progenote with an RNA genome [5,6,7,11,12,13,14,15,16,17,18,19]. The first scenario for many years was the hegemonic scenario in the scientific community; however, the accumulation of data from several organisms has raised some questions about this scenario. The first of these refers to the distinct constitution of the membranes of the organisms of the basal lineages, Bacteria and Archaea. The data referring to the formation of the membrane, along with its route of synthesis, lead to a more parsimonious scenario of independent origin of the membrane in these lineages, making a scenario of the monophyletic origin of the cells untenable, discarding, at least temporarily, this scenario in the origin of cellular organisms [18]. The same questioning can be applied to the second scenario because in this scenario it is also suggested that before the diversification of the basal cellular lineages, the emergence of cell systems had already occurred and that these first lineages derived from this pre-existing one. Regarding questions about the first scenario, the suggestion that this organism already had a DNA genome is opposed to the fact that the genome replication machinery of Bacteria and Archaea does not show homology, and therefore, it is more parsimonious to think that these systems arose independently in both lineages [5,6,7,8,9]. In this context, the most parsimonious scenario before the emergence of cellular lineages is the last scenario, where biological systems still functioned in a semi-open organization and with information stored in RNA molecules. Thus, let us assume this scenario to think about the origin of the DNA genome in the basal lineages.

The results of the present study allow us to suggest that cellular lineages emerged independently, even with an RNA genome. In this scenario, viral lineages emerged in parallel with cellular lineages. In the bacterial lineage, the emergence and maturation of DNA-dependent DNA polymerases, and of the DNA genome, occurred after initial diversification of this group, which occurred in at least two independent ways. In the case of family A, the transition process occurred in the ancestral lineages of mitochondria, wherein this machinery was transferred to viral lineages that spread to the rest of the bacterial lineages. In the case of family C, the replication machinery and DNA genome that emerged in another bacterial branch were transferred to a viral lineage and then spread to the rest of the bacterial lineages. Through this route of maturation of the DNA genome, the machinery emerged and established itself within cellular lineages. Viral lineages of bacteria inherited this characteristic of cellular lineages and acted as a dissemination system for the rest of the lineages that had not yet made the transition from an RNA genome to a DNA one. 

**Figure 4:**
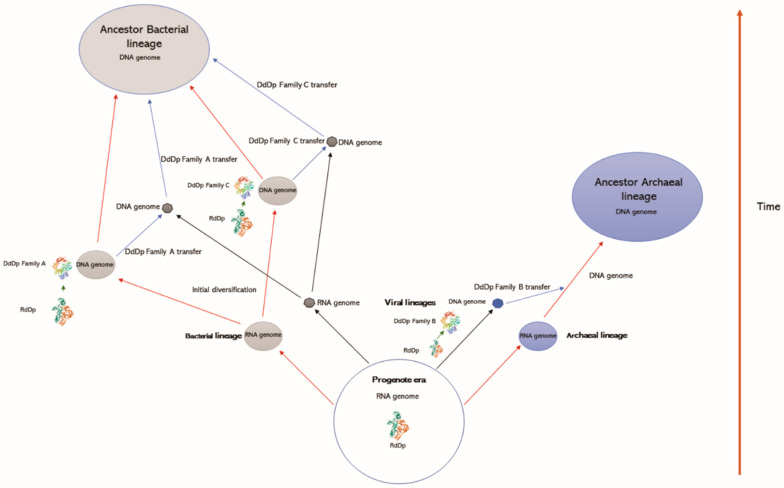
Diagram of the evolution of DNA polymerases in bacteria (family A and C), archaea (family B), and viruses. Red arrows indicate the events of origin and diversification of cellular lineages. Black arrows indicate the events of origin and diversification of viral lineages. Blue arrows indicate lateral gene transfer events.

In the case of family B, the data suggest that the transition from an RNA genome to a DNA genome occurred first in the viral lineages and was transferred to the basal archaeal lineage before the diversification of this group. Thus, we suggest that DNA in Archaea arose as the main informational molecule before the same process occurred in bacterial lineages. In this context, we assume that all families of DNA-dependent DNA polymerases had an RNA-dependent DNA polymerase as their ancestral molecule through a process of diversification by radiation (Figure 4). 

This fact may explain in part why there is not any homology across the different families. As each family followed its own evolutionary path, the similarities in terms of sequence were lost, leaving just the structural similarities essential to this group of proteins’ functionalities. Although we assume the scenario described above, other scenarios were proposed and discussed by Leipe et al. [8] and by Edgell and Doolitttle [41]. Three hypotheses were put out by these authors to account for the variation in cellular DNA polymerases. (i) The bacterial and archaeal/eukaryotic replicative systems have evolved from the LUCA replication apparatus, and the main replicative enzymes are homologs but have diverged rapidly, and in several cases, beyond recognition. (ii) The LUCA possessed both a bacterial-type and an archaeal/eukaryotic-type DNA replication system (one of these could be responsible for repair), and the existence of two radically different systems in extant cells is due to differential gene loss in the bacterial and the archaeal/eukaryotic lineages. (iii) Either the bacterial or the archaeal/eukaryotic replication system is the direct descendant of the ancestral replication apparatus, whereas the other version evolved by recruitment of non-homologous proteins, accompanied by replacement of ancestor components. Our data do not allow any of these scenarios to be discarded; however, here we assumed an a priori scenario that allowed us to consider the events that occurred according to the latter hypothesis to be plausible. The attempt to explain such ancient events cannot be seen as a trivial and definitive analysis. 

As a result, while the identification of the enzymes participating in certain processes may not directly point to the process’s origin, it may provide us with hints that help us build hypothetical scenarios. In this sense, creating many scenarios is legitimate for generating new hypotheses that need to be evaluated through improvements in data and analysis methodologies, even though they do not reflect a final decision on the matter.

Clearly, the findings presented do not put an end to discussions concerning the origin and evolution of the DNA genome in cellular lineages; rather, they introduce a fresh scenario for discussion considering potential present-day and future evidence.

## Data Availability

Data is contained within the article or supplementary material.

## Supplementary Materials

The following supporting information can be downloaded at: https://www.mdpi.com/article/10.3390/v15030749/s1, Sequence Data: Sequences used in the generation of phylogenetic trees for families A, B and C; Original Phylogenetic Tree: Original phylogenetic tree for family A, B and C.
