# Supplementary figures and images for: Natural History of DNA-Dependent DNA Polymerases: Multiple Pathways to the Origins of DNA
Source: Viruses. 2023 Mar 14;15(3):749. doi: 10.3390/v15030749 (PMC10052633; doi:10.3390/v15030749)

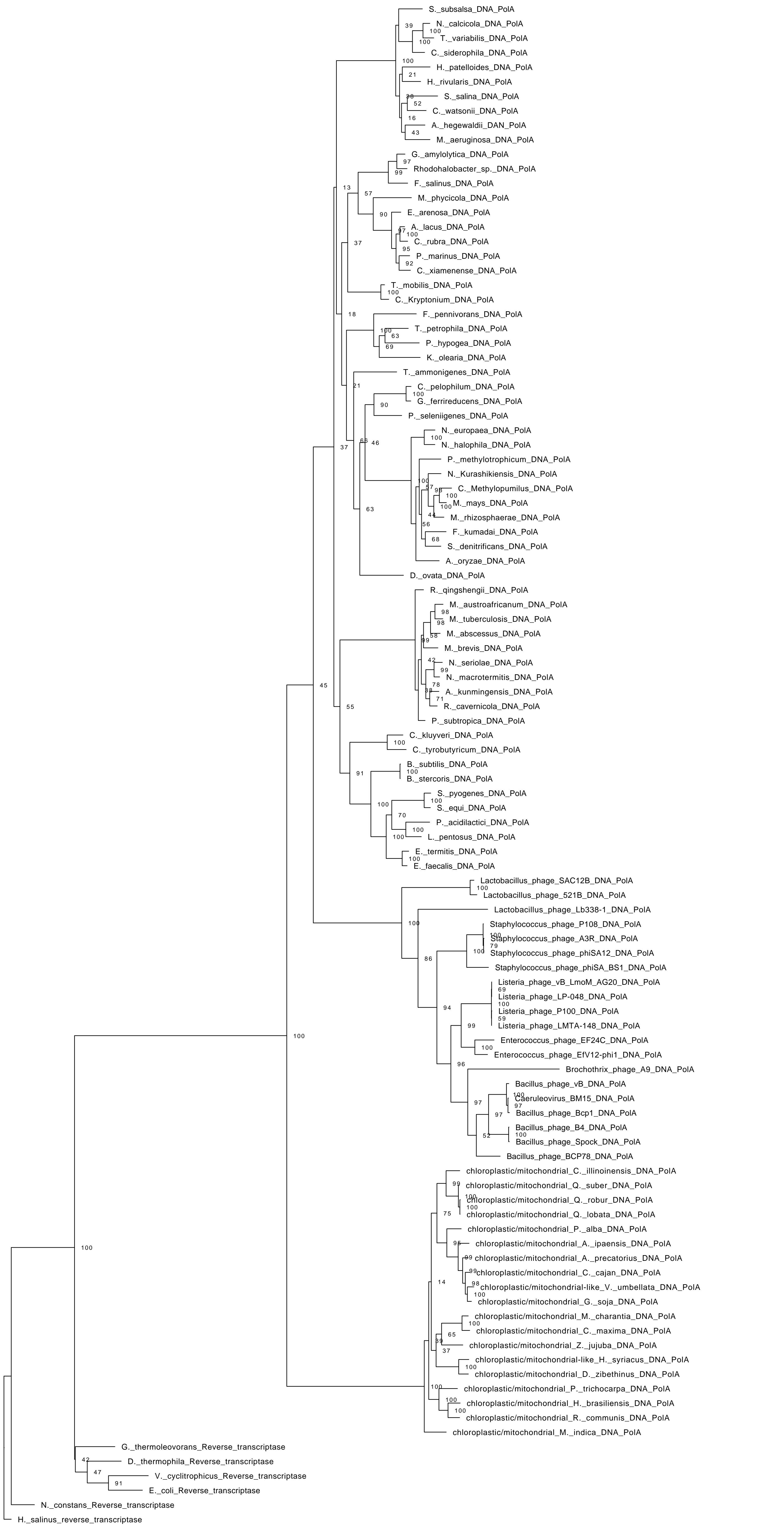

Supplement: Supplementary file 1 [file viruses-15-00749-s001.zip › Tree DNA Polymerase Family A.pdf]

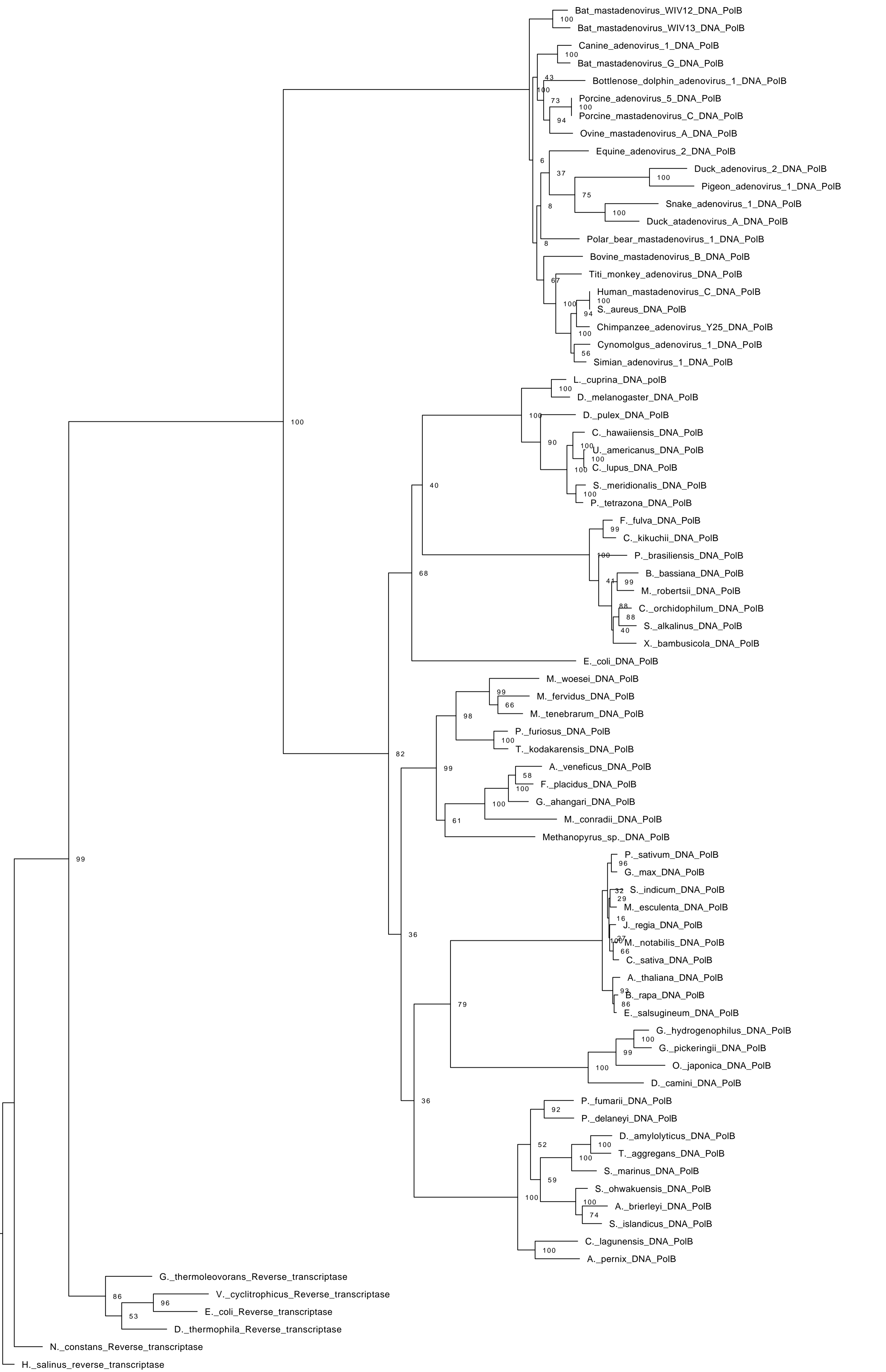

Supplement: Supplementary file 1 [file viruses-15-00749-s001.zip › Tree DNA Polymerase Family B.pdf]

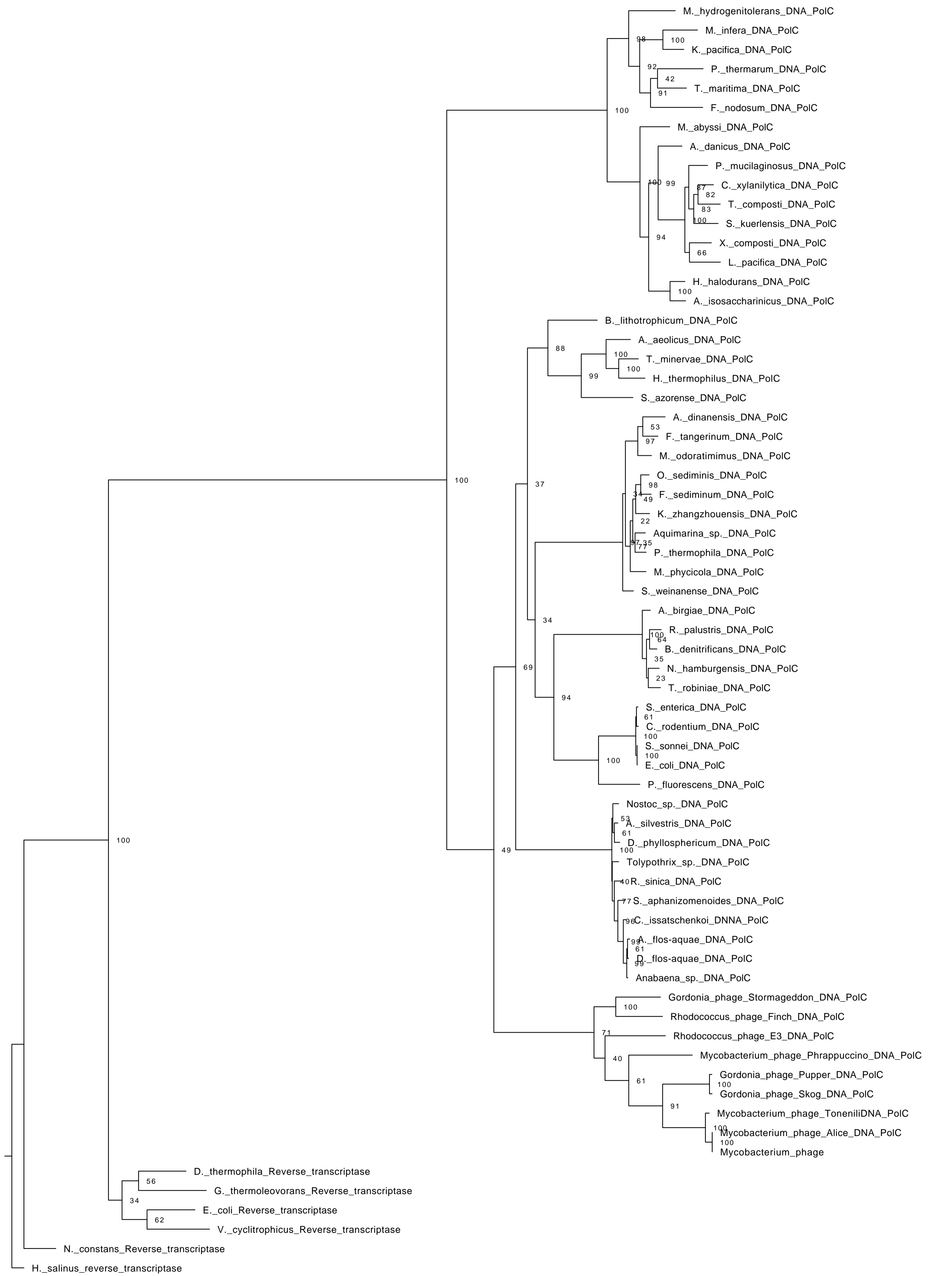

0.8

Supplement: Supplementary file 1 [file viruses-15-00749-s001.zip › Tree DNA Polymerase Family C.pdf]
